# A Review on Graphene-Based Light Emitting Functional Devices

**DOI:** 10.3390/molecules25184217

**Published:** 2020-09-14

**Authors:** Muhammad Junaid, M. H. Md Khir, Gunawan Witjaksono, Zaka Ullah, Nelson Tansu, Mohamed Shuaib Mohamed Saheed, Pradeep Kumar, Lee Hing Wah, Saeed Ahmed Magsi, Muhammad Aadil Siddiqui

**Affiliations:** 1 Department of Electrical and Electronic Engineering, Universiti Teknologi PETRONAS, Seri Iskandar 32610, Perak, Malaysia; pradeep.hitesh@gmail.com; 2 Department of Electronic Engineering, Balochistan University of Information Technology, Engineering, and Management Sciences, Quetta 87300, Balochistan, Pakistan; saeed_19001716@utp.edu.my (S.A.M.); muhammad_18003606@utp.edu.my (M.A.S.); 3 BRI Institute, Jl. Harsono RM No.2, Ragunan, Passsar Minggu, Jakarta 12550, Indonesia; Gunawan.witjaksono@gmail.com; 4 Center for Photonics and Nanoelectronics, Department of Electrical and Computer Engineering, Lehigh University, 7 Asa Drive, Bethlehem, PA 18015, USA; tansu@lehigh.edu; 5 Department of Mechanical Engineering, Universiti Teknologi PETRONAS, Seri Iskandar 32610, Perak, Malaysia; shuaib.saheed@utp.edu.my; 6 Flexible Electronics R&D Lab, MIMOS BERHAD, Technology Park Malaysia, Kuala Lumpur 57000, Malaysia; hingwah.lee@mimos.my

**Keywords:** graphene, graphene oxide, CNTs, SWNT, light source, thermal emission, plasmons-assisted emissions, electroluminescence, excitons, trions

## Abstract

In recent years, the field of nanophotonics has progressively developed. However, constant demand for the development of new light source still exists at the nanometric scale. Light emissions from graphene-based active materials can provide a leading platform for the development of two dimensional (2-D), flexible, thin, and robust light-emitting sources. The exceptional structure of Dirac’s electrons in graphene, massless fermions, and the linear dispersion relationship with ultra-wideband plasmon and tunable surface polarities allows numerous applications in optoelectronics and plasmonics. In this article, we present a comprehensive review of recent developments in graphene-based light-emitting devices. Light emissions from graphene-based devices have been evaluated with different aspects, such as thermal emission, electroluminescence, and plasmons assisted emission. Theoretical investigations, along with experimental demonstration in the development of graphene-based light-emitting devices, have also been reviewed and discussed. Moreover, the graphene-based light-emitting devices are also addressed from the perspective of future applications, such as optical modulators, optical interconnects, and optical sensing. Finally, this review provides a comprehensive discussion on current technological issues and challenges related to the potential applications of emerging graphene-based light-emitting devices.

## 1. Introduction

Graphene is a single layer honeycomb structure of carbon lattice [1] with many interesting behavior and characteristics [2]. It is considered the most promising material for engineering design due to its extraordinary electrical, mechanical, and chemical properties [3,4,5]. Since 2004, the exfoliation of single-layer graphene [6] has been investigated and explored in every field of science. Additionally, an exponential increment in terms of publication numbers was perceived with substantial results [7,8]. Graphene as an attractive material has been extensively researched in many fields of electrical and electronic engineering, such as touch screens, light detectors, transparent conductors, photovoltaic cells, and energy systems [9,10,11]. Besides, the optoelectronic properties of graphene-based materials are being highly investigated for the application of optoelectronic devices [12]. The direct bandgap opening [13,14,15], strong light-matter interaction [16], photoluminescence [17], electrons field emission [18], and the evidence of emission radiation from graphene make it a promising material for the future generation of optical devices to produce a thin, flexible, and lightweight optoelectronics device [19].

In the context of light-emitting diode (LEDs), the existing LEDs technology is considered quite mature, even at the consumer end. LEDs have already been applied in several fields, including signage, display backlight, general illumination, and communications [20,21]. LEDs have high-performance characteristics, such as low power consumption, high efficiency, high-speed response, low operating voltage (< 4 V), current (< 700 mA) characteristics, and small outline dimensions (< 10 mm to 10 mm) [22]. Solid-state light-emitting devices (LEDs) are classified into two main streams: organic (OLEDs) and inorganic LEDs. Conventional inorganic LEDs are composed of brittle or hard powdery material, i.e., silicon, phosphor, lens, and glass. Many key research areas in LED designing, such as quantum efficiency of the active region, current-flow design, resistive losses, electrostatic discharge stability, and optimization of luminous flux per LED package, need improvements [23]. However, flexible and transparent light-emitting devices with a small footprint are of key focus. The development and manufacturing of OLEDs carried out in recent years are still facing drawbacks [24]; for example, the panel fabrication at high-temperature conditions and to incorporate flexible substrate PET (polyethylene terephthalate). ITO-based electrodes are too stiff for the development of flexible OLEDs [25]. The graphene is also considered an alternative material to ITO due to excellent electrical conductively, transparency, and chemical and thermal stability [26]. Graphene and doped graphene, such as transparent electrode [27], with nanowire [28], CNTs, and SWCNTs [29], have also been studied with improved LEDs performance in term of current spreading enhancement [30] and ohmic contact formation with reduced growth temperature [31]. 

Moreover, the light emission from different graphene structures, such as single and multilayer graphene [32], reduced graphene oxide [33], graphene nanoribbons [34], and quantum dots [35,36,37] has been reported. The emission radiation in spectral range (NIR) near-infrared to the visible region (VIS) and grey body radiation from electrically drive graphene-based devices have also been demonstrated practically. Several theoretical attempts have also been made to justify the light emission from graphene and related structures, where the corresponding emission is explained by the thermal emission radiation [38], plasmons assisted emission [39], and electroluminescence [40]. Besides, the light emission from graphene was also demonstrated with numerous potential applications, such as light-emitting devices, sensors, bioimaging, drug delivery, optical modulators, and optical interconnects [41,42,43]. 

Thermal emission from electrically driven graphene devices was also reported in the spectral region from infrared (IR) to visible range [44]. Thermal emissions from the graphene layer were ascribed to local heating, with almost the entire spectrum of grey body radiations, where a small fraction of energy about (~10^−6^ part) is converted into light emissions [32,45]. The sustainable high current density (10^7^ A.cm^−2^) in micron-sized CVD graphene as compared to conventional tungsten filament (~100 A.cm^−2^) and with low thermal mass three times smaller in the magnitude of silicon cantilever offers the prospect of high-frequency operation. Likewise, there is constant demand for the development of new IR sources with low cost, safe, and portable safe gas sensors, particularly for mine security. The existing IR sensors use conventional incandescent light sources with several limitations such as lifetime, wavelength, time response, excessive power consumption, and the requirement of explosion-proof casing in a flammable environment. MEMS-based electromechanical silicon emitters, as an alternative IR source, also exhibit low response time up to ~100 Hz modulation frequencies [46]. Solid-state LEDs offer more advantages, particularly in terms of higher modulation speed. However, the radiative efficacy of the LED operated in infrared is limited by the non-radiative Auger recombination [47]. The infrared emission from LED, an intrinsic process, mainly depends on charge carrier density and particularly on narrow semiconducting bandgap. The combination of narrowband semiconductors with a higher refractive index was used for the fabrication, which binds the photon escaping mechanism and limits overall efficiency [48]. However, the demonstration of thermal emission from a large-area graphene layer coupled with extraordinary thermal conductivity offers a prospect for the high-efficiency IR light source. 

The realization of ultrafast plasmons-based optical signal source at the nanoscale is considered as a longstanding goal, the potential of the graphene-based emitter to revolutionize optoelectronics, thus allowing ultrafast optical signal processing for communication [49]. When the electron beam is exposed to the optically excited surface plasmons of graphene, the unidirectional, chromatic, and tunable emission from IR to X-ray was realized from the graphene [50,51,52]. The theoretical investigation and experimental demonstration of this mechanism predict the existence of plasmons at VIS and IR wavelengths [53]. Besides, the plasmons-assisted light emission from graphene in VIS, and even shorter wavelength was illustrated by the interaction of surface plasmons and charged particles [50,54]. Significantly, the 2D quantum Čerenkov effect (ČE) can also be achieved in graphene, due to the unique properties of high field confinement, surface plasmons, and low phase velocity. The quantum ČE effect in 2D graphene refers to the emissions when shockwave plasmons are excited by the hot carrier in manners as in three-dimensional (3D) medium. The 2D quantum ČE leads to light emission from the VIS to the IR region, where surface plasmons are coupled as photon radiation due to impurities or roughness in graphene structures [55]. 

**Scheme 1:**
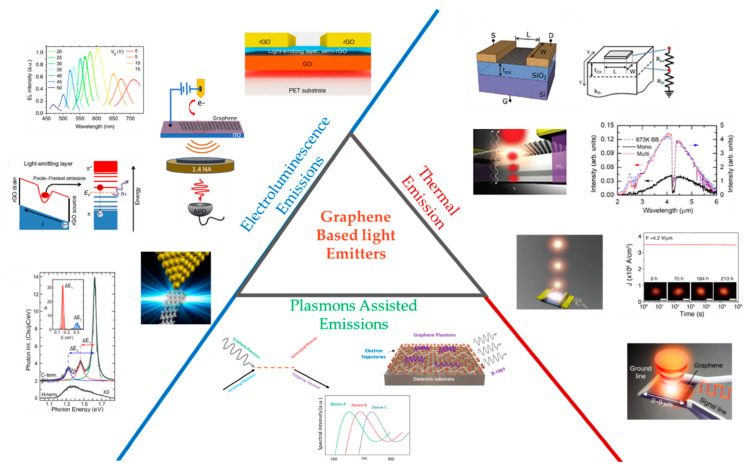
Graphene-based light-emitting functional devices, where the emission radiation from graphene has been ascribed to thermal, electroluminescence, and plasmons assisted emissions [58,60,61,62,63].

Graphene can also produce the luminescence effect by inducing an energy bandgap. Therefore, there are two possible ways to induce bandgap in graphene: the first is by cutting it into ribbons or quantum dots, and the second is by chemical or physical treatments by connectivity reduction of the π-electron network [56]. The electroluminescence (EL) effect observed from graphene and graphene-related structure is quite interesting, as graphene can be used as an active material for light-emitting devices. The phonon-assisted EL emission in the VIS region was also reported from the electrically biased graphene supported on a substrate [57]. The VIS emission from graphene was also demonstrated by the excitation of electron tunneling current in STM (scanning tunneling microscope) using a voltage biased tip, which is attributed to hot electroluminescence [58]. In addition, the tunability of the EL emission spectrum for the entire visible spectrum was demonstrated by the application of gate voltage, which is quite challenging in the modern solid-state (LEDs) industry [59]. Lastly, graphene-based light emitting devices classified based on the light-emitting mechanisms shown in Scheme 1. 

In this article, we have extensively reviewed the recent progress in graphene-based light-emitting devices, including device structure, fabrication, and their optical and electronics properties related to device structure, material, and emission mechanism. The manuscript is subdivided into four sections based on the light emission mechanism from graphene-based light-emitting devices, where the light emissions are ascribed to thermal, electroluminescent (EL), and plasmons assisted emissions. We have also highlighted the future applications of graphene-based light emitters, such as a broadband light source for optical modulators, optical interconnect, and broadband spectrum light source for gas detection. This article also discussed the current technological issues and challenges related to the recent development of graphene-based light emitters.

## 2. Graphene-Based Thermal Emitters

The high carrier mobility [64,65,66,67] and high thermal conductivity [68,69,70] of graphene make it a candidate material for future high-speed optoelectronic devices. For the design and development of graphene-based light-emitting devices, the non-equilibrium process of electron-hole combination is not efficient, because of the zero bandgap nature of graphene, due to the rapid relaxation of electron-phonon and electron-electron interaction [64]. On the other hand, the high strength, high current densities (10^9^ A/cm^2^ in nanoribbons) [71], particularly high current density 10^7^ A/cm^2^ reported in micron-sized graphene fibber synthesized by chemical vapor deposition (CVD) [72], with higher temperature stability enables broadband thermal emission from the graphene [32]. When compared to the 100 W (current density ~100 A/cm^2^) tungsten filament light bulb, the graphene is as an attractive potential material for the development of incandescent light source [73]. Besides, most of the existing incandescent infrared sources suffer from several shortcomings, such as limited wavelength range, lifetime, and sensitivity to flammable gasses. In addition, as an alternative infrared source, the microelectromechanical systems (MEMS) based silicon thermal emitters depict relatively slow response (~1 kHz maximum modulation frequency) because of their slow responsivity characteristics with few mV/W [74]. Besides, the fundamental limitation of semiconductor-based light-emitting diode (LED) has the advantage of high modulation speed at IR (infrared) ranges [75]. Significantly, the thermal emissions (grey body radiations) demonstrated from graphene-based structures with its low mass, and its excellent electrical conductivity holds promise for the future generation of infrared sources [76,77,78]

**Figure 1:**
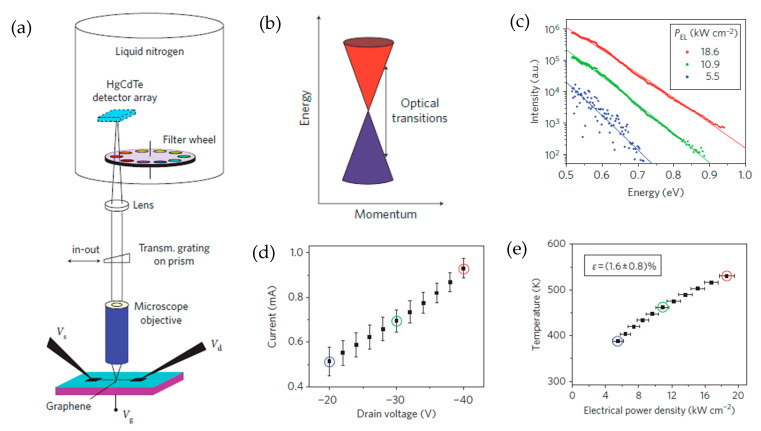
(**a**) Shows the experiment setup comprised of infrared imaging, vacuum prob station, and microscope with sensitivity between 0.95 and 0.5 eV. In this, the grating transmission was utilized for spectroscopy and then detached for infrared imaging; to cut down the dark count, a low pass filter was utilized. (**b**) The direct point vicinity related to the electron dispersion. (**c**) The measured intensity of thermal infrared radiation from biased graphene under three power levels, where the slide line agrees with grey body radiations, fit to Equation (1). (**d**) Shows I-V characteristics curve and the error bars were utilized because of slight hysteresis response observed in measurement, where, the values of red, green, and blue highlighted bars correspond to the electrical power level. (**e**) shows the temperature as a function of electrical power level, based on grey body fitting [45].

Characteristics of emissions and performance of light-emitting devices are strongly influenced by their thermal behavior, whereas distinct features of the biased graphene layer, such as spatial resolved thermal emissions, high carrier densities, and spatially located direct points can be utilized for extraction of temperature-dependent distributions. Besides, the electrically derived graphene layer depicts the gate-dependent location of a hot spot. Though the fixed location of temperature maxima was also reported, the infrared thermal emission from graphene provides an appropriate and noninvasive characterization tool for graphene-based devices. Marcus Freitag et al. reported spatial resolution thermal emission radiation from the graphene-based transistor, which can be utilized to obtain the carrier densities, temperature distribution, and spatially resolved location of the Dirac point in the graphene layer. Moreover, the applied gate voltage can also be utilized to control the temperature maxima and the corresponding location of the stationary hot spot, where the thermal emissions in the infrared region depict a non-invasive and convenient characteristic tool for graphene-based devices [45]. To analyze thermal emission from the graphene, the back-gate FET has been fabricated from the mechanically exfoliated graphene. When the current passes through the biased graphene layer, the electrical energy transformed into joule heating, which dissipates mainly through the substrate and the metal contacts [79]. The detailed experiment has been demonstrated in Figure 1a, where a small fraction of applied electrical energy—about ~10^−6^—was transformed into emission radiation and detected with the spectral resolution near-infrared region. Graphene shows high thermal conductivity at room temperature (K_Gr_ = 5,000 W m^−1^ K^−1^) [80], where the generated heat is carried out into metallic contact.

It has also been reported that the Umklapp scattering reduces heat dissipation at higher temperatures [81], and the scattering of surface polar phonon enhance the energy dissipation through the SiO_2_ substrate [82]. Moreover, the heat dissipation through substrate dominates for the devices having channel length more than a few millimeters. The infrared emission spectrum was observed to be fit with grey body radiation by modifying the emissivity value to 1 (see Figure 1c):

(1) u(v,T)=ε8πh2c3exp(hvkBT−1),

where *T* is the temperature, *hv* the photon energy, and *u* is the density of spectral energy. The equivalent drain to source currents and voltage are shown in Figure 1d. Figure 1e shows the extracted temperatures, Figure 1c is well-fitted with grey body radiation. A grey body like a response from graphene was also observed with constant emissivity, with +20% deviation order values, possibly limited by uncertainty response of the optical system instead of the emissivity graphene itself [45]. The thermal emission radiation from graphene has been limited to the near-infrared region, and the performance limitation was imposed due to dominant heat dissipation through the substrate. Significantly, the cooling of hot carriers has been ascribed to the dominating extrinsic scattering from polar surface phonons trapped charge carriers and impurities.

**Figure 2:**
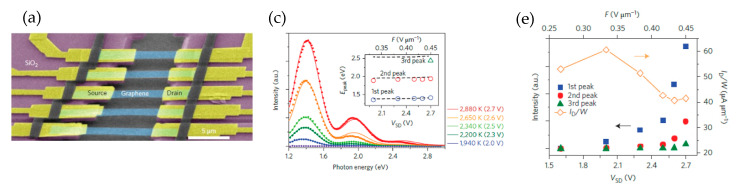

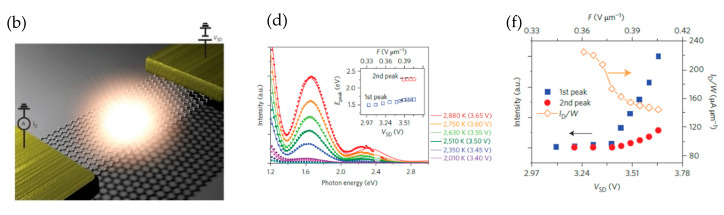
Light emission from suspended graphene: (**a**) shows the single-layer suspended graphene structure, (**b**) illustrates the light emission (hot spot) from the center of suspended graphene at room temperature at (< 10^−4^ Torr). (**c**,**d**) The emission radiation peaks from the suspended graphene-based structure under applied electric field, (**f**) as a function of V_DS,_ where the dotted line presents the calculated energy of the emission peaks related to the interference effect of thermal radiations. (**e**,**f**) The integrated intensity referred to the individual emission peak, (**e**) tri-layer, where I_d_ and V_DS_ correspond to the applied electric power and electric field, respectively, as with the increase of V_DS_, the applied electric power is decreased, whereas the rapid increase in emission intensity was observed [60].

Furthermore, the bright and visible light emission from a suspended-like graphene structure was also reported by Y.D. Kim and coworkers. The mechanically exfoliated graphene, low pressure, and plasma-assisted CVD grown single-layered graphene (on Cu foil) were deposited on a SiO_2_/Si substrate, for the realization of device fabrication. The experimental setup for light emission from suspended graphene (trench-based structure) on SiO_2_/Si substrate under the high electric field is shown in Figure 2a,b (under vacuum conditions < 10^−4^ Torr). Therefore, the significant reduction in heat transport from suspended graphene structure was observed [83], where hot electrons (~2,800 K) were accumulated at the center of the graphene layer and consequently enhanced 1K fold efficiency of thermal radiations. When V_DS_ exceeds the threshold value, the suspended graphene begins to emit light. The light-emitting area and brightness were observed to be propositional with V_DS_. The light-emitting spot was observed in the middle of the suspended graphene, and it overlaps with the position of maximum temperature. Moreover, reproducibility and stability of light emission have been experimented under vacuumed conditions. Besides, the light emission spectra with several peaks were observed from suspended graphene (with a trench depth of 900 to 1,100 nm) ranging from 1.2 to 3 eV as depicted in Figure 2c (monolayer) and Figure 2d (trilayer). Furthermore, several peaks of light radiation were also reported from suspended graphene configuration with a trench depth of 800 to 1,000 nm. It has also been determined that the intensities of the light emission peaks depend on the trench depth D and are independent of the number of graphene layers. The interference effect was also observed among the emission radiation from graphene and reflected light from the substrate. The relationship between two successive destructive interferences and energy septation was established in Equation (2):

(2) $$∆\;\left(D\right)=\frac{1,239.8\;nm}{2D}\;eV,$$

According to Equation (2), D ≈ 1,000 nm for Δ ≈ 0.6 eV agrees with results (Figure 2c,d). The substrate supported graphene under a high electric field; the electrons are in equilibrium with optical phonons at temperatures of up to ~2,000 K. However, the acoustic phonon and optical phonon are not in equilibrium because of the decaying rate of the optical phone. The decay rate of optical phonons to the acoustic phonons is much lower as compared to optical phonons to electron-hole pairs [84,85]. Moreover, in the case of the suspended graphene structure, the associated lattice temperature of acoustic phonons was higher as compared to the graphene stacked on the substrate. Therefore, the generated heat could not dissipate through the substrate [84], whereas the electrons and optical phonons were induced at higher temperatures, as shown in Equation (3) [86]:

(3) $$T_{op}\left(\alpha \right)=T_{op}+\alpha \left(T_{op}+T_{o}\;\right)$$

Moreover, the electrically biased layered graphene facilitates the accumulation of spatially localized hot electrons (~2,800 K), which makes graphene an ideal material for the application of light-emitting device at the nanoscale. In addition, the tunability of broad-spectrum emission can be attained by manipulating the strong interference effect; particularly, the interference effect in an ultra-thin and flat layer of graphene could allow the realization of unique flexible, thin, transparent and large-scale display modules [61]. In the case of freely suspended graphene, the issues of the dominant heat dissipation through the substrate can be overcome. The graphene-based suspended structures are more promising for bright, visible, and efficient light emission radiation with a broad spectrum. Specifically, the emitted light from the suspended graphene structure interacts with the surface of the underlying substrate and produces an interference effect. The interference effect between the emitted light and reflected light from the substrate could be further utilized for the tuning of wavelength emission. However, this proposed design represents excessive heat dissipation with low efficiency [87]. 

**Figure 3:**
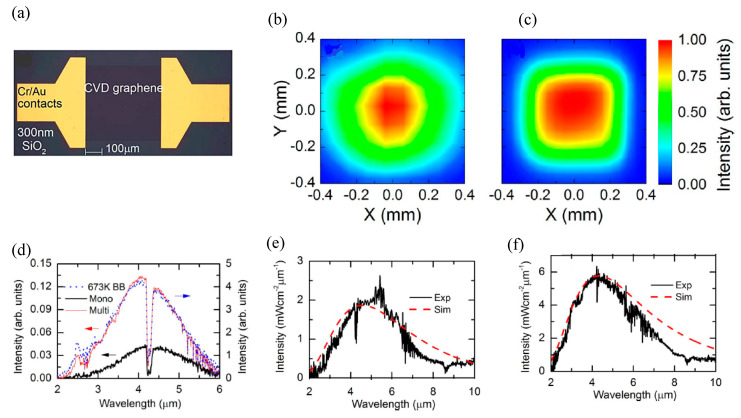
(**a**) The device fabricated with large area graphene 0.5 × 0.6 mm, transferred on 300 nm SiO_2_ substrate, with CR/Ar source and drain contacts. (**b**,**c**) shows the thermal emission from single and multi-layered graphene, (**b**) emission from single-layer graphene with peak current injection of 44 mA, (**c**) emission from multi-layer graphene with peak current injection of 52 mA. (**d**) The emission spectra of single and multi-layered graphene, calibrated with the back body at 673 K, where the peak emission was approximately 4 um and the large deep trench at 4.2 µm was ascribed to the CO_2_ absorption. (**e**,**f**) shows the measured and simulated spectra with a dash [61].

Thermal emission from large-area, single, and multilayered graphene, coupled with extraordinary conductivity, has been highly demanding for the development of nano-scaled high-frequency infrared sources. In this context, Lawton and co-workers have also investigated the thermal infrared emission from large-area CVD graphene. The device was fabricated by transferring the single and multi-layered graphene on a highly doped SiO_2_/Si substrate. The large area graphene was defined by using electron beam lithography and Ar/O_2_ reactive ion etching, where the source and drain contacts were deposited by utilizing Au/Cr thermal evaporation, as presented in Figure 3a. Furthermore, typical layered graphene was synthesized via CVD, with the resistance of 1750 and 1300 Ω, measured at low current (1 mA) for monolayer and multilayer graphene, respectively [88]. It was also reported that for both single and multilayer of graphene, the resistance quenches with increasing temperature [89]. Thermal emission measured by using the microscopic scanning system; the low noise signal amplifier along with a lock-in amplifier was utilized for the sensitive phase measurement. The thermal emission from single and multilayer graphene (under zero gate bias voltage) with peak current values of 44 mA and 52 mA is shown in Figure 3b,c, respectively. The light radiations from single-layer graphene were also observed in the form of a single hotspot with large areas [46]. Specifically, the thermal emission was found to be dominated by the joule heating effect, where the hot spot was observed in the middle of the graphene channel wherein the gate leakage prevents the movement of a hot spot as the function of applied gate voltage.

However, in the case of a single layer, graphene emission is found to be more consistent with channel resistance and exposed to the whole area. The light emission from the hotspot is proportional to the square of the applied current, which indicates joule heating. Specifically, the thermal temperature of light-emitting graphene has been well-defined by the duration of the current pulse and dimension of the graphene channel. In the experimental description, the emission spectral was calibrated with a black body source at 673 K as shown in Figure 3d, where the calculation-based emission spectra were presented in Figure 3e,f. Besides, the difference in estimated and measured emissivity was 2% and 6%, where it was expected to increase linearly with the increase of graphene layers [46]. The graphene-based thermal emitters are also considered a promising light source in the near-infrared region due to the low emissivity of 2.3% per layer [90,91,92]. 

**Figure 4:**
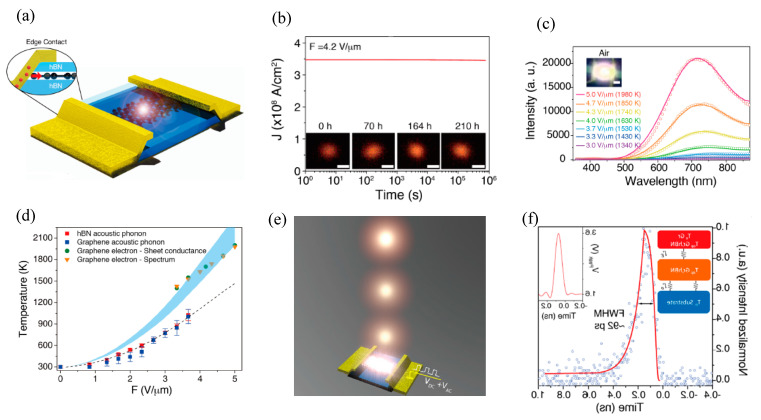
Ultra-fast thermal emission from hBN encapsulated graphene layer (**a**) the fabricated device, where it consists of a single-layer graphene encapsulated with hBN from both sides and edges are connected with source and drain contacts. (**b**) The response of light emitter under static electric field F = 4.2 V/um depicts insignificant variations in the intensity of light emission and current density. (**c**) The response of light emitters under different values of electric field and electric power under the vacuumed condition and air, respectively, under high electric response F = 5.0 V/um, peak emission of 718 nm near-infrared is observed. (**d**) Demonstrates the electronic temperature and derived temperature together under high biasing conditions, the T_ap_ of hBN and graphene are almost equal; however, it is lower than the electronic temperature. (**e**) The pulse response from ultrafast light emitter under electric control, where light pulses are verified by the time associated with single-photon counting, (**e**) The ultrafast light pulses (92 ps) from graphene-based light emitters, where solid blue line depicts electrically drive pulse of 80 ps, which refers to the bandwidth of 10 GHz. (**f**) The red line corresponds to the thermal radiations and transient temperature exponential fit where schematic at the left-top side presents the energy relaxation time in the graphene layer [96].

Moreover, the graphene-based thermal emitter with large area characteristics depicts a relatively low modulation speed of 100 kHz as demonstrated under steady-state conditions [93]. Young at el. also investigated the transient properties of the graphene-based thermal emitter, where ultrafast thermal emission was demonstrated from the hBN/Graphene/hBN heterostructure. The dry van der Waals pick method has been utilized to assemble heterostructure on SiO_2_ (285 nm)/Si substrate by transferring the exfoliated monolayer graphene and hBN flakes. Followed by etching of the graphene-based heterostructure, the Cr/Pd/Au metal was deposited for the realization of source and drain contacts, as depicted in Figure 4a. The heterostructure based on the 2D material layering of graphene and hBN present the charge carriers’ mobility close to the scattering rate of intrinsic acoustic phonons at room temperature [94]. In addition, the heterostructure structure with a clean interface at the atomic level assembled by the staking the multiple layers of 2D material is essentially required for the reduction of extrinsic effects such as defects, charge impurities, roughness, and blisters due to aggregation [95]. Therefore, due to the stability and high optical phonon energy of the hBN, visible light emission has been observed under the conditions of a high current density of ~4.0 × 10^8^ A/cm^2^, a high electric field up to ~6.6 V/μm, and with zero gate voltage.

Additionally, remarkable stability has also been reported from encapsulation (with hBN) edges contact even under high current density, high electric field, and temperature. The light emission spectrum under vacuum conditions and in air is shown in Figure 4a,b, respectively. The emission spectrum extends from 400–1600 nm (visible to infrared) with an emission peak of 720 nm, and from another device, the emission peak of ~1000 nm was also measured. Particularly, the emission spectrum has remained unchanged in open-air conditions. Notably, the higher intensity emission peak at 720 nm from encapsulated graphene with hBN could be ascribed to the dielectric optical cavity (with refractive index *n* = 2.2). The dielectric cavity formed by the formation of the hBN layer on a substrate, which can be utilized to tailor the thermal radiation by modification of the optical density of states [97]. From the encapsulated graphene, the maximum Te = 1980 K was achieved under the F = 5.0 V/μm electric field, as shown in Figure 4b. 

The radiation enhancement of 460% was also observed with a peak at 720 nm as compared to the grey body radiations (from graphene). Likewise, the reduction in emissivity (absorption) has been observed in the near-infrared regime, which was ascribed to the Pauli blocking. The current saturation was also reported under the high electric field for the hBN encapsulated graphene [98]. The current saturation can be attributed to the emission of optical phonon or the efficient backscattering, either from the substrate or graphene [96]. Figure 4d shows an illustration of the resulting temperature together with electronic temperature, where under the high electric field, the T_ap_ both in hBN and graphene is almost the same but below the electronic temperature. Figure 4e shows the ultrafast light pulses from the encapsulated graphene under electric control, and the output pulse 92 ps width was reported, which infers to the bandwidth > 10 GHz (see Figure 4f). Besides, the long-term performance of the device has also been tested under high current density (J ≈ 3.4 × 10^8^ A/cm^2^), electric field (F = 4.2 V/μm), and in vacuum space (~10^−5^ Torr). The persistent performance of the device without any substantial degradation has been reported for ~10^6^ s (see Figure 4f), where the suggested lifetime under 50% current degradation was ~4 years.

**Figure 5:**
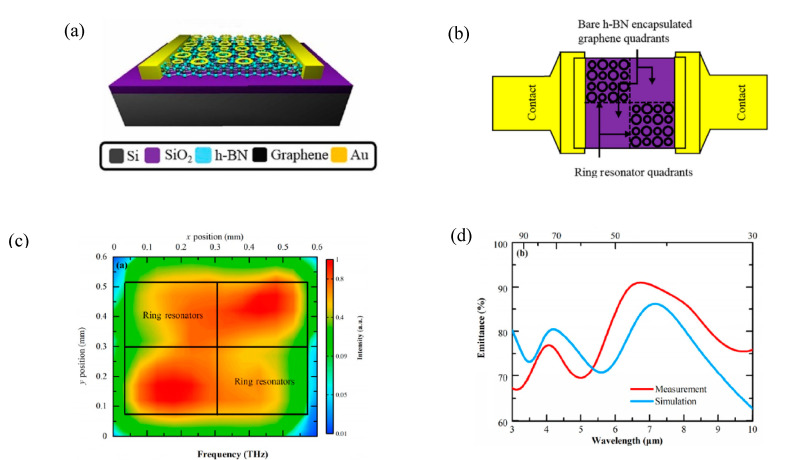
(**a**) The schematic diagram of metamaterial and graphene-based thermal emitter. (**b**) The top view metamaterial graphene-based thermal emitter for the direct comparison of the light-emitting surface patterned with four quadrants (with each 250 μm × 250 μm). The ring resonators consist of 5/50 nm thick Cr/Au, patterned on the encapsulated graphene surface, (**c**) the spatial measured thermal radiations from the fabricated device. (**d**) The experimental (red line) and simulated (blue line) emission spectra [104].

It has also been reported that the emission spectra can also be manipulated by the using sub-wavelength photonic crystals, where the enhancement in the narrowband thermal emission from Tungsten filament was achieved by the resonant coupling modes of 2D crystals [99,100]. Besides, the metamaterial also provides a versatile platform for the manipulation of electromagnetic propagations [101]. In recent studies, Cheng et al. demonstrated (mid-infrared) duel band thermal emissions from the hBN encapsulated graphene layer with the help of a frequency-selective surface metamaterial. A thermal emitter with high modulation speed comprises of metamaterial as a frequency selective surface. The metamaterial surface was comprised of concentric ring resonators rings, which was used to tune the emission radiation further into two discreet bands, as shown in Figure 5a. The dual-band emission can be utilized in the application of gas detection. Mainly, the high modulation frequencies of 71 THz (3.6 μm) and 43 THz (7 μm) obtained from the fabricated device can be used for gas detection, which corresponds to CO_2_. The conductivity of graphene can be modeled as a two-dimensional ohmic sheet as in Equation (4) [102,103]:

(4) $$\sigma_{d}=\frac{i\;e^{2}k_{b}}{\pi \hbar^{2}\left(\omega +i2\Gamma \right)}\;\left[\frac{\mu_{c}}{K_{b}T}+2\ln\left(e^{-\frac{\mu_{c}}{K_{b}T}\;}+1\right)\right]+\frac{i\;e^{2}}{4\pi }\;\ln\left(\frac{2\left|\mu_{c}\right|-\left(\omega +i2\Gamma \right)\hbar }{2\left|\mu_{c}\right|+\left(\omega +i2\Gamma \right)\hbar }\right),$$

where the right term in Equation (4) contributes to the inter and intraband transition, and the variables *µc* and *Γ* are chemical potentials and carrier scattering with the graphene layer. Furthermore, the first emission wavelength band was used as a reference, whereas the second wavelength band resonates with related absorption of the targeted gas being detected. The frequency and wavelength of the emission radiation correspond to the size of the circle resonators. The resonance frequency of the corresponding emission can be calculated by Equation (5):

(5) $$f=\frac{nc}{2\pi r\;\sqrt{\varepsilon_{\mathrm{eff}}{}_{\;}}},$$

where *n* is the mode number, *c* is the velocity of light in vacuum, and *r* is the averaged value for the outer and inner radius of the resonating ring, $\;\varepsilon_{\mathrm{eff}}\;$ is a value of the effective permittivity corresponding to the metamaterial, which is extracted from the *S-*parameter calculations. The thermal energy radiation from the encapsulated graphene was observed upon the joule heating effect, whereas the embedded ring resonators on the frequency selective metamaterial surface resonate in phase with a resonant frequency. Moreover, enhanced emission radiation was observed due to the constructive interference of resonators rings with thermal radiation. Specifically, the destructive interference at other frequencies due to the out-of-phase resonance will reduce thermal emissions. For the direct comparison of thermal emission from graphene with and without metamaterial, the fabrication process was divided into four quadrants (250 μm × 250 μm), depicted in Figure 5a. The ring resonators comprise of the 5 nm, 50 nm, Cr, and Au metal thick layer. The two quadrants in diagonal direction were decorated with ring resonators, and the off-diagonal quadrant was kept without ring resonators. The thermal emission with spatial variations is plotted on the logarithmic scale, depicted in Figure 5b,c, where red and blue lines correspond to the experimental and simulated results, respectively. 

**Figure 6:**
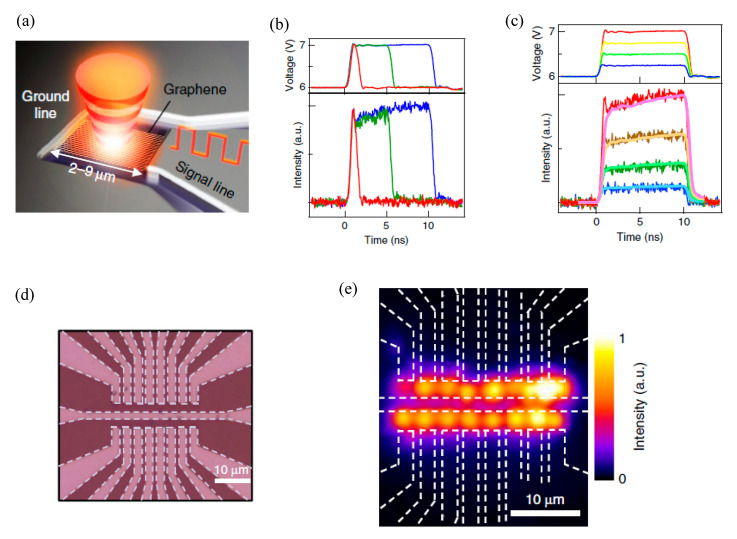
(**a**) The structure of the graphene emitter black body emitter encapsulated with Al_2_O_3_, the CVD graphene was deposited on the square SiO_2_/Si substrate, which is attached with source and drain electrodes, where high modulation optical signal obtained by applying input signals. (**b**) Shows time resolve emission from the device under different pulse width (1, 5 and 10 ns) and voltage amplitude (6–7 V), (**c**) the time resolve emission from the device with a pulse width of 10 ns and amplitude 6 to 7 V. (**d**) The optical image of the highly integrated graphene 2 × 8 arrays thermal emitter with CVD graphene, with size 2 × 2 μm and pitch 3 μm. (**e**) NIR camera image of the light emission from the arrayed device, each array is connected to the same voltage (10 V), and series resistance is introduced with each array to demonstrate the relative uniform light emission from each device [62].

The integration of conventional light-emitting sources for the semiconductor-based integrated system is a significant challenge due to their exertion of direct growth on the silicon-based substrate [105]. Yusuke and co-workers reported the highly integrated, high speed (with ultrafast modulation) on-chip graphene-based thermal emitter for optical communication via remote heat transfer. Fast response of ~100 ps was reported, which corresponded to the higher modulation speed of ~10 GHz achieved experimentally from single and few layers of CVD graphene. Figure 6a exemplifies the structure of the device under DC-biased conditions, with broad-spectrum emissions in the near-infrared region. The spectral emissions, including telecommunication wavelength obtained via the microscopic image of the two-dimensional array emitter, are demonstrated in Figure 6b, where the emission intensity increases proportionally with applied voltage. The light-emitting graphene layer was capped by the Al_2_O_3_ insulator using ALD (atomic layer deposition) to inhibit the oxidation of the graphene layer. The conductance of the capped graphene layer is reduced to 50% because of the scattering of charged impurity and the scattering of surface polar phonons [106]. The corresponding temperature of the graphene layer linearly depends on the applied voltage due to the effect of the electron scattering with phonon induced by the joule heating [107] and maximum temperature of 750 K at V_DS_ = 8 V was observed. The time-resolved emissions depend on the amplitude and width of the input rectangle pulse. It shows the quick response time of ~0.4 ns, which dominates the signal generator with response time ~0.5 ns, as shown in Figure 6a,b. The fabrication of a two-dimensional 2 × 8 array device shown in Figure 6d,e demonstrate the relatively uniform light emission from the two-dimensional arrayed device. The encapsulated emitter with Al_2_O_3_ can operate for more than 100 h [62]. Finally, all graphene-based thermal emitters discussed in Section 2 are summarized in Table 1.

## 3. Plasmons-Assisted Emissions from Graphene

There is increased interest on highly integrated optoelectronic devices with surface plasmons polarities and nanoscale light emitters [108]. In recent studies, it has been demonstrated that the ability of graphene plasmons (GPS) can be utilized as a platform for strong light-matter interaction [109,110,111]. Furthermore, the dynamics of highly confined light with tunable GPS makes the graphene an extremely promising candidate for the design of light emitters at the nanoscale [112,113]. Besides, the strongly-confined and high momentum graphene plasmons can enable the development of tunable, monochromatic, highly directional, and high frequency (10^14^–10^15^ Hz) light-emitting sources with relativity low energy electrons [114]. Additionally, the high-quality light emitter with a small footprint with X-ray and extreme ultraviolet radiation is extremely exciting in the research perspective of medical engineering and natural science. However, the graphene plasmons-based short-wavelength emitter has not been investigated, as compared to other graphene-based promising applications [115]. 

**Figure 7:**
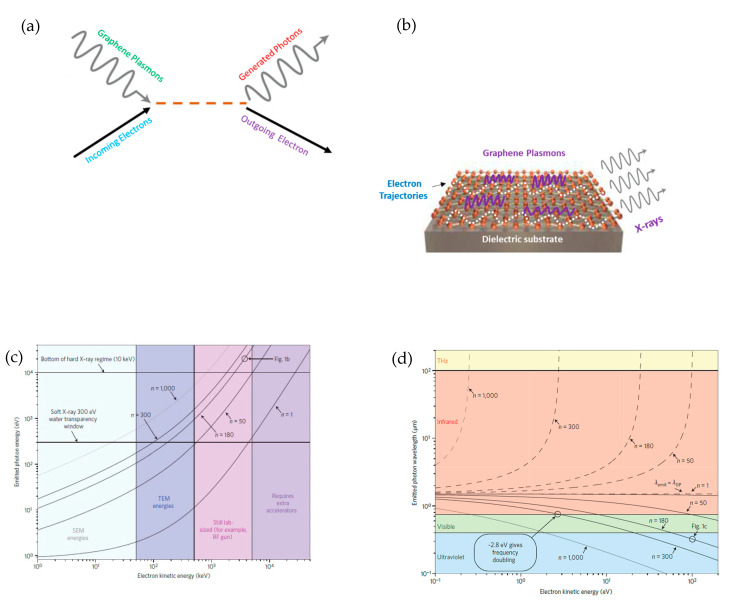
Graphene plasmonic-based source for short wavelength radiation. (**a**) shows the electrons-plasmon interaction, where the white dotted line demonstrates the free electrons interacting with plasmons glowing with blue and red bars, (**b**) illustrates the process of graphene plasmons emission from hot carriers. (**c**) The soft and hard X-ray radiation from graphene-based free electrons radiation source was achieved without any acceleration stage, with various frequency conversion region, the lines correspond to confinement factor (*n* = 1,50… 1,000) where *n* = 1 is shown as a reference. (**d**) shows the up-converting (black line) and down convection (dash line) of related frequency from graphene-based free electrons source. It is observed that the coupling of phase velocity of graphene plasmons velocity and electrons velocity cause the down-conversion of frequency [116].

Besides, the tunable, monochromatic, and highly directional light emission from the graphene layer with the interaction of electrons and plasmons has been reported by Liang and colleagues [116]. The schematic diagram of the graphene plasmon-based radiation source is shown in Figure 7a,b. The generation of highly directional X-ray emissions from modestly relativistic electrons is presented, which does not require additional neutron shielding. Moreover, the low energy electrons are possibly generated in a device on-chip for the frequency conversion mechanism. In design configuration, the graphene sheet was staked on a dielectric substrate with a grating structure, wherein the dielectric substrate was utilized to sustain graphene plasmons. The graphene layer was excited by coupling a focused beam when the electron beam was launched in parallel with the surface of the graphene. The consequent interaction between the graphene plasmons field and low energy electrons induce transverse electrons oscillations [116]. Therefore, soft and hard X-ray radiation from the graphene surface was accomplished without any further acceleration stage; the various frequency conversion regions are shown in Figure 7c,d. Specifically, the plasmons are quasiparticles interacting with modestly relativistic electrons, which govern by the electron-phonon interaction, the same as fundamental rules for the radiation process. However, different results have been reported because the graphene plasmons generate much higher momentum than the energy of photons at the present state. Additionally, graphene plasmons have longitudinal field components, which photons do not have. Consequently, the electron-plasmons scattering was different from the electron-photon scattering, as stated by the standard Thomson or Compton effect [116]. 

In related research, Pavel A. Cherenkov showed light radiation from charged particles when the charge moves faster than the phase velocity of light in the medium. However, the requirement of relativistic partials makes light emission unreachable at nanoscale optoelectronic devices [117]. Notably, the existence of high-velocity hot carriers (Fermi velocity ~10^6^ ms^–1^) is possible, even in graphene sheets larger than 10 μm large [118]. However, the plasmons in graphene show extremely low phase velocity (a few hundred times slower than the speed of light) [119,120]. Therefore, the frequency matching between plasmons and hot carriers is possible via electrical excitation, which enables the high rate of GPs emissions. In addition, the propagating charge carrier inside the 2D graphene layer could efficiently excite GPs via the Cˇerenkov emission (CˇE) process. Significantly, higher rates of GPs emission have been observed in CˇE than previous studies on phonons/photons [119,121]. 

Additionally, the surface plasmons create energy levels higher than 2E_F_, which exceed the energy level of photon emission and enable plasmons emission from terahertz to infrared and possibly invisible emissions spectra. Likewise, the tunability of energy levels from the implication of external electrical excitations can improve radiation parameters such as direction, spectrum, and intensity. More significantly, the emission radiation behavior of graphene-based material such as high-frequency radians [32,64,122], saturation current [98,123], black body radiations [124], and tunable spectrum [125] can be explained by the following transition phenomenon. The quantum Cherenkov effect can be defined as the process of spontaneous emission from the charger carrier emitting into graphene plasmons, which can be calculated by Fermi’s golden (GPs) [126,127]. In related studies, Ido Kaminer et al. developed the quantum Cerenkov theory for plasmonic emission radiations and analyzed the phenomenon of novel Cerenkov-induced plasmonic emission. The graphene plasmons can provide a platform to overcome limitations related to relativistic particles for plasmonic emitters through the high field confinement and low phase velocity. 

**Figure 8:**
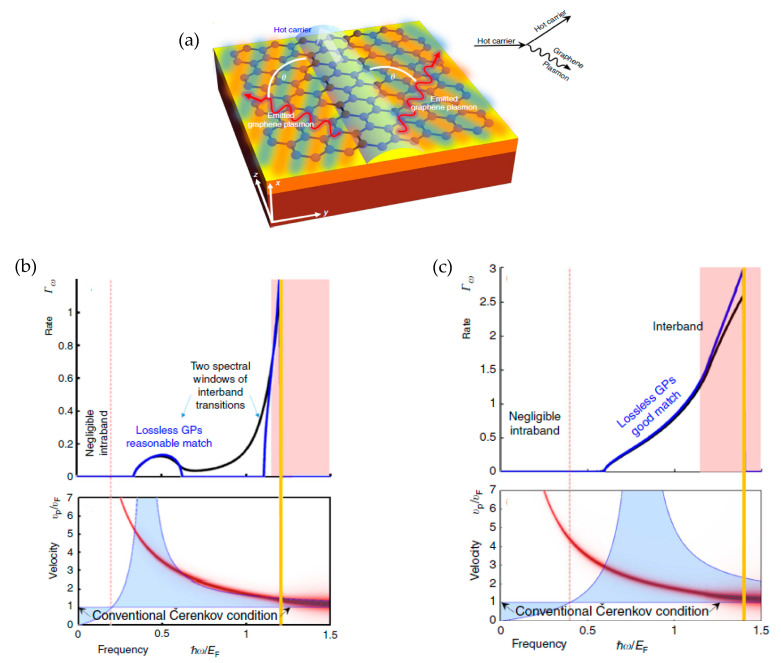
The sketch illustrates the 2D Cerenkov emission process in graphene. (**a**) GP emission from the graphene layer by the inner flow of hot carrier, diagram demonstrating the GP emission-related process due to the hot carrier in the graphene layer. (**b**, (**c**) illustrates the spectrum of the CˇE GP emissions, the red region corresponds to the GP losses and emission losses presented in black, where emission approximation is depicted with blue. The orange line shows the spectral cut off because of the Fermi sea, where all available states are filled. In the lower part of (**b**) and (**c**), the red curve depicts the GP related phase velocity, and its thickness corresponds to the GP losses [55].

Moreover, the coupling of plasmons and charge carriers inside the graphene layer enables the highly efficient two-dimensional Cerenkov emission, where versatile, tunable, and ultrafast conversion from electrical signal source can be used to overcome the limitations. Figure 8a shows the graphene plasmon emission from a hot carrier inside the graphene. The white arrow shows that the hot carriers make a transparent blue shape, which excites graphene plasmons, as shown in red; blue bars propagate along the graphene surface on a substrate marked in orange, red, and yellow. The Cerenkov angle with which graphene plasmons are emitted is denoted by Ѳ and defines the wiggling red arrows in the *z*-axis, which is in the direction of the hot carrier motion [55].

According to the classical approximation, the charge carriers outside the graphene layer satisfy the $\hbar \omega \;\ll E_{i}$ condition, exactly as [126]. However, the charged particle moving inside graphene shows much lower energy due to the existence of massless fermions, which modify the conventional threshold velocities and allow the phenomenon of CE. Figure 8 demonstrates interband and intraband CE, where charge velocities are lower than the conventional threshold velocity. More significantly, most of the radiation was emitted backward, which was considered impossible in other materials [128]. The CE spectral distribution of CE interband and intraband transitions over the angular degree of freedom is shown in Figure 8b,c. The spectral range with a non-vanishing blue line has been demonstrated with several spectral cuts. 

**Figure 9:**
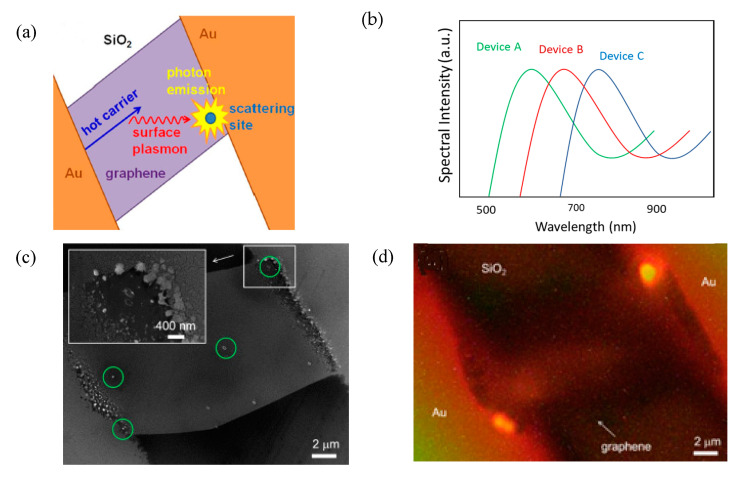
(**a**) The schematic diagram of graphene light-emitting FET based on CE effect. (**b**) The graph shows the light emission spectral intensity versus emission wavelength for the three different devices A (green) (V_G_ = 30 V, V_DS_ = 7 V), B (red) (V_G_ = 0 V, V_DS_ = −30 V), and C (blue) (V_G_ = 40 V, V_DS_ = 18 V), with different channel lengths. The curves are well fitted to the Gaussian function presented with black lines. (**c**) SEM image of the device; the white rectangle shows the light-emitting area, the defected area of the graphene channel is highlighted by green circles. (**d**) The SEM image corresponds to emission from scattering sites [76].

Several theoretical studies reveal that visible and short-wavelength emission spectrum can be attained through the graphene layer by the interaction of surface plasmons and charged particle [55]. However, Beltaos et al. reported experimental studies on visible and near-infrared light emission from a graphene-based field-effect transistor. The possible light emission from a mechanically exfoliated graphene-based channel (~15–25 µm) on Si/SiO_2_ (~300 nm) substrate was explained by the CˇE effect. The phenomenon of plasmonic coupled emission from GFET is further investigated by using spectroscopic technique and imaging. The light emission from the electrically excited graphene layer was observed as a yellow-orange spot located near the edge of the graphene channel near Au electrodes (see Figure 9a). Likewise, the emission parameters (spectral intensity, wavelength) can be controlled by the applied drain, source, and gate voltage. Notably, the emission spectra were highly affected by the channel length and formation defect in the graphene layer. The corresponding emission spectra of GFET fabricated with different channel sizes ~15, 20, and 25 µm, correspond to the A, B, and C devices, respectively (see Figure 9b). Under the positive and negative value of applied voltage (V_DS_ and V_G_), similar behavior from each device was observed, which implies that both charge carriers and current directions can be utilized for light excitations. Exceptionally, the physical location of the light-emitting spot was highly influenced by the scattering sites, nanoparticle, edges, and formation defects. However, it was also demonstrated in optical microscope color images of electrically excited devices that the position of light-emitting spot remained independent of the applied variation of V_DS_ and V_G_. The SEM image as shown in Figure 9c,d depicts the light emission location near the edge along the graphene channel, where the defect in the graphene layer and light-emitting spot is marked by the green spot. Notably, the emission has been observed near the vicinity of the defect near Au nanoparticles and edge, which supports the hypothesis of controllability of light emission from scattering sites. Finally, all of the plasmon-assisted emissions of the graphene-based emitters discussed in Section 3 are summarized in Table 2.

## 4. Electroluminescence (EL) Emissions 

Graphene has attracted plenty of attention from the perspective of optoelectronic applications due to its amazing optical and electronic properties, such as massless fermion, direct cons, tunable fermions, and nearly flat absorption [129,130,131]. In recent studies, photo-electrons conversion has been reported for carbon-based material such as graphene [132], carbon nanotube [133], quantum dots [134], and graphene-based plasmonic [135] photodetectors. However, recent studies have also been highly focused on photo-electrons conversion, whereas the inverse process of electro-photon (electroluminescent-EL) conversion in the graphene layer can also provide new visions into carrier dynamics. Moreover, the graphene in intrinsic form is a gapless material, but the electroluminescence requires a material with additional energy states for electron-hole combination. Therefore, graphene can be modified physically or chemically to induce bandgap [136,137]. The bandgap in the graphene layer can also be induced by the substrate and the tip induces an electric field [138]. Moreover, the change in the Fermi level of the graphene has also been observed from graphene staking on substrates with different work functions [138]. Particularly, the EL effect has also been reported from graphene-based materials. The EL footprints have been demonstrated from single to a few layers of graphene structure [57] such as carbon nanotubes [139], carbon nanoribbons [140], and quantum dots [141].

### 4.1. Electroluminescence (El) from Graphene

**Figure 10:**
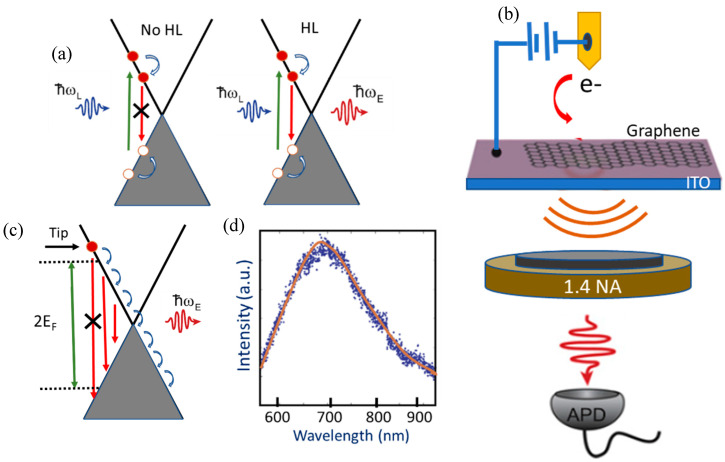
STM hot luminescent from graphene. (**a**) The diagram demonstrates the hot luminescence from graphene. In the case of undoped graphene when a photon with energy ($\hbar \omega_{L}$) used excite hole-electron pair, no hot luminescent observed, because of the non-radiative combination of the electron-hole pair. However, in the case of doped graphene with enough bandgap, the hot luminescent is observed with radiative decay of electron-hole combination. (**b**) The experimental setup is demonstrated in the diagram with the doping mechanism of graphene due to the substrate and tip. (**c**) The schematic shows the hot luminescent process in graphene; the grey shaded area shows the energy level < 2E_F_ required for radiative decay. The electrons with > 2E_F_ energy induce non-radiative decay presented with a dashed arrow. (**d**) The electroluminescent spectra from single-layer graphene under V_B_ = 3 V [58].

Only a few studies have been explored EL emissions from graphene, where the EL measurement was either limited to the few-layered graphene or nano-scale photonic crystals [142]. In related research, Ryan Beam et al. reported STM (scanning tunneling microscope) induced hot electroluminescence from single-layer graphene at ambient conditions. Explicitly, the emission radiations depend on the number of graphene layers and the relative energy of the injected carriers. In the case of pristine graphene, the electron-hole pair can be excited by the incident photons [143], where the non-radiative combination of electron-holes occurs in the valence band due to the unavailability of energy states [144]. However, in the case of the doped graphene layer, the incident photon can produce a radiative effect due to the presence of the energy states [145]. Therefore, the EL effect requires a doping level within a limit range of $\hbar \omega_{L}>2\left|E_{F}\geq \;\hbar \omega_{E}\right|$, where $\hbar \omega_{L}$ and $\hbar \omega_{E}$ are the relative photon energy of laser emissions [146]. Moreover, EL emissions from graphene can be transformed into the STM vision by replacing incident photons with electron injection, as demonstrated in Figure 10a. Moreover, the energy of injected charge carriers can be controlled by applying base voltage V_B_; the experiment setup is illustrated in Figure 10b. Besides, in the experimental setup, initially, the glass substrates were cleaned with plasma oxygen and then coated with ITO, where the P-type graphene layer with a maximum bandgap of ~0.5 eV was obtained through the doping effect of contact leads. 

In addition, the change in the Fermi energy of the graphene layer can be due to two possible reasons: first, the high work function of the A_U_ tip material, which raises the P-doping level, and secondly, the bias voltage V_B_, which can also induce the capacitive effect. These two possible doping effects can induce a bandgap from ~0.5 to 0.65 eV [147]. The induced bandgap in graphene is enough for an electroluminescent effect at near IR and visible emissions. Likewise, the electroluminescence effect was experimented in the graphene layer for energies level ≤ 2E_F_, as shown in Figure 10c with the grey shaded area. Besides, if the injected carrier has energy less than 2E_F_, then electrons lose their energy before the process of radiative decaying. The output radiation spectrum for single-layer graphene is shown in Figure 10d.

The established LEDs-based technology is the key component of today’s solid-state semiconductor industry [148], where the existing LEDs technology has enabled plenty of applications, such as low cost, high-efficiency performance, smart display, and high-performance communication. However, the tuning of light-emitting spectra in LEDs’ design is quite challenging; the emission spectrum of LEDs is predefined in the fabrication process [149]. In related research, Wang et al. reported a spectrally tunable and visible light emission from the graphene-based heterostructure structure device, as shown in Figure 4a. The light scribe surface of the semi-reduced GO nanoclusters is interfaced between the GO and reduced GO. The zero-bandgap nature of rGO and the extremely large bandgap between (π and π*) bandgap of GO implies that the luminescence in rGO and GO is not possible, though, the experimentally observed broad PL feature is a strong signature of the presence of localized states in the semi-reduced GO layer, similar to those in rGO quantum dots [150]. The charge carrier mobility in the semi-reduced GO network approaches 10 cm^2^V^–1^S^1^ that supports the carrier injection. Moreover, the emission spectrum can be controlled by the doping level or by the gating level from blue to red (∆ ≅750 nm). Significantly, the devices fabricated with the light scribe method shows visible light emission with a high brightness up to 6,000 cdm^−2^, but short lifetime and low efficiency ~1% [63].

**Figure 11:**
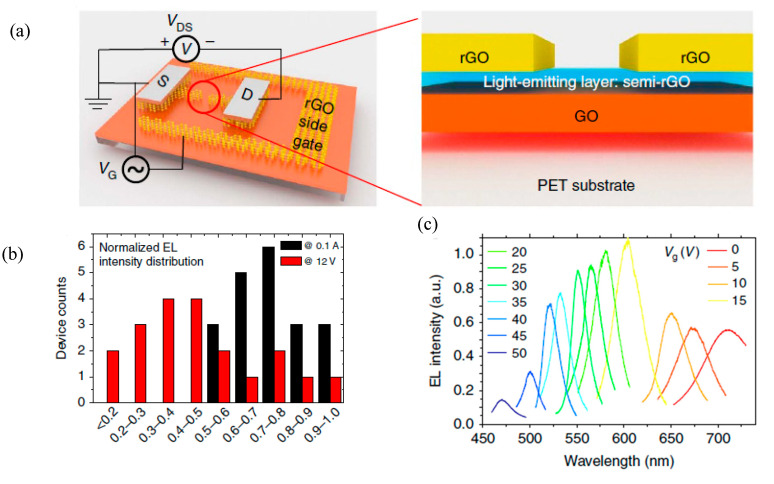

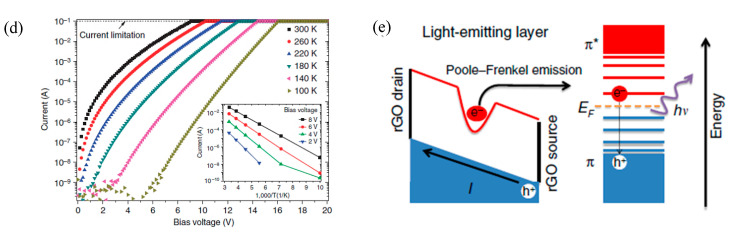
(**a**) The schematic of GFLED, the interfacing layer with semi-reduced GO (blue) between GO (orange) rGO (yellow) is responsible for the light emission from GFLED, (**b**) shows a histogram for the distribution of EL versus fixed voltage and current values, (**c**) EL response of GFLED under gate voltage from 5 to 50 V. (**d**) The voltage biasing response of GFLED verses source to drain current at different temperature conditions, (**e**) illustration of the Poole-Frenkel emission, the excitation of a trapped electron in unoccupied lowest energy state and the excitation of electron combination with p band holes for the photon emissions [63].

Moreover, the light emission intensity as a function of the drain to source bias is shown in Figure 11b. Particularly, EL intensity can be controlled by the driving current; a relatively uniform device operation was reported at fixed current bias 0.1 A (I_max_/I_min_ < 2). The EL spectral can be tuned from light blue to dark red emission spectrum by the adjustment of the Fermi level, where the implication of side gate voltage was used to tune the Fermi level. Besides, the ambient doping can also be utilized for the tuning EL spectra. The variation in EL spectral under different gate voltage values is demonstrated in Figure 11c, where wavelength shifts from 690 nm (V_G_ = 0) to 470 nm (V_G_ = 50 V) were observed. The temperature-dependent charge transportation was also analyzed by plotting drain-source current versus gate voltage (I-V), as shown in Figure 11d. The I-V relationship found to be fit with measured conductivity in the Arrhenius Plot shows the electrons injection in system charge emission manners. More specifically, the linearity feature in log(I) plot versus V_1/2_ indicates an agreement with Poole Frankel’s current law [151]. The Pool Frankel effect is defined as the movement of trapped electrons from the coulomb binding state to the conduction band [152]. Figure 11e illustrates the Pool Frankel emission of the graphene-based FELED, where the high density of states in the oxygen-rich light-emitting layer work as charge traps states. Besides, in a high electric field, the low energy states’ occupied charges at discrete energy levels are excited to perform thermally generated electron-hole combination, which results in EL. The zero bandgap nature of graphene limits its use as an active material for optoelectronic applications [153]. However, outstanding properties of graphene nanoribbons (GNRs) combined with tunable bandgap make it a promising candidate for the development of future light-emitting sources.

### 4.2. Electroluminescence (El) from GNRs

**Figure 12:**
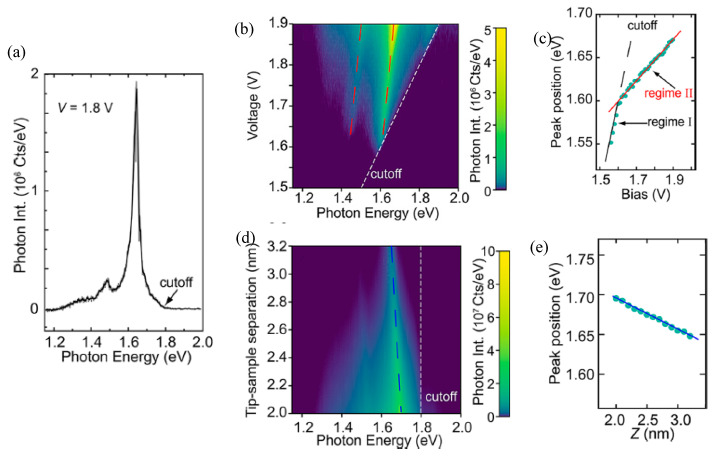
(**a**) Shows the electroluminescence spectrum of C-terminated GNRs. (**b**) and (**d**) show the characteristics of STM emission and voltage dependence (z = 2.3 nm) and tip distance (V = 1.8 V); for above the threshold voltage V > 1.53 V, no emission is observed. Above the threshold voltage, both vibronic and central peak is observed. At higher voltage (V > 1.9 V), no further spectral features were observed. (**c**) and (**e**) confirm the shift in emission line as a function of Z and V, respectively [140].

Several theoretical and experimental studies have been explored on the related energy bandgap of GNRs (graphene nanoribbons) with design configurations [154,155], where optical characterizations such as time-resolved, Raman, reflectance difference, and UV-VIS/IR spectroscopy, have been addressed [156,157,158,159]. Nevertheless, experimental studies on SWNTs-based light-emitting devices are very limited; so far, as only a few assembled measurements with weak and featureless emission spectra were reported [34,160]. In the same context, Chong et al. reported electroluminescence from suspended GNRs between the substrate and gold (Au) capped tip of STM. The bright and narrowband emission of red light is obtained from the GNRs junction, where the emission spectra can be tuned via the biased voltage applied at the junction, but it lies below the bandgap of infinite GNRs. The Ab initio calculation reveals that the emission implicates the localized energy states at GNR termini [140], where the emission spectra of GNRs strongly depends on the nature of GNRs and STM tip connection. In the case of covalent bonding, a sharp and intense luminescent peak (40 m eV) was observed, whose energy can be controlled by the distance between tip and surface as well as applied voltage. Inferring to unbiased GNRs junction on Au, significantly lower transition energy (~1.16 eV) is obtained in comparison with an optical bandgap of ~2 eV [161]. The STM-LE emission is shown in Figure 12a. The existence of vibrionic features and sharpness of emission spectrum are mismatched with earlier studies on thermal [32] and plasmonic emissions [66,162], where intrinsic transition radiations are highly suggested from suspended GNRs. The characteristics of the electroluminescence spectrum from GNRs are shown in Figure 12b,d. The display of the plot in Figure 12c,d confirms the energy shift as a function of the applied voltage. Comparatively, the emission radiation process stayed with a low quantum efficiency (10–5 photon/electron), where the ability of the suspended GNRs withstand high current (~4 nA), allowing the high-intensity light emission of 10^7^ photon/s (~1 pW at 30 nA). Moreover, the intensity of light emission from GNRs was found to be 100 times higher than that of the plasmonic emission reported from a single-molecule optoelectronic device [163].

### 4.3. Electroluminescence (El) from FLG

**Figure 13:**
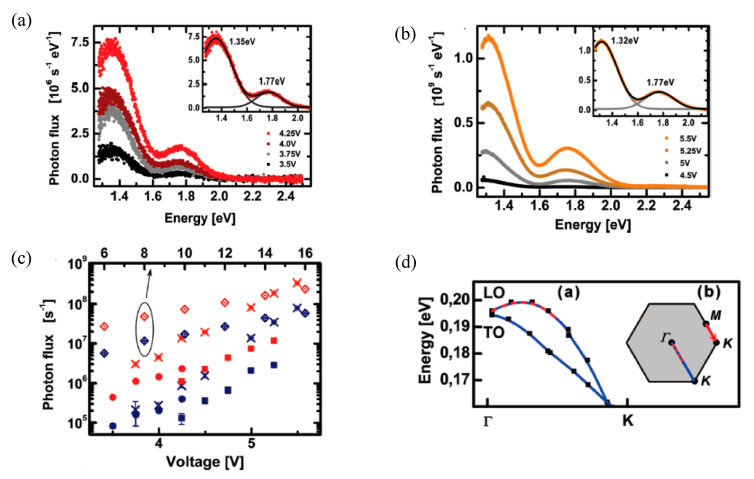
(**a**) and (**b**) show the phonon-assisted electroluminescence spectra from SWNT, with diameter from 0.94 to 1.49, (**c**) the relation between phone flux and biased voltage has been demonstrated, where the blue and red sign present relative peaks of low and high energy emissions. (**d**) The phonon dispersion along Γ-K direction and Brillouin zone with its symmetry point M, K, and Γ. The momentum for the electronic transition from M to K (red arrow) provided by phonons in the Γ-K direction (blue line). In the relevant momentum range (red dashes), phonons of the L_O_ branch have energy consistent with that required to explain the electroluminescence data [57].

Essig and coworker reported phonon-assisted electroluminescence from a few-layer graphene (FLG) structure and metallic SWNT. The visible spectrum with peaks at 1.4 and 1.8 eV was reported from SWNT (diameter range of 0.7 to 1.5 nm); the comparable response was found in multiwall carbon nanotubes. The light emission spectra from SWNT and their evolution with applied voltage is illustrated in Figure 13a,b. Moreover, the spectral photon flux *d_n_/(dt dE*) (photons/second) and electron-volt were calculated from intensity I (count per second) as in Equation (6):

(6) $$\frac{d_{n}}{dt\;dE}=I\frac{s}{∆\lambda }\;\frac{hc}{E^{2}},$$

where Δ*λ* is the bandwidth, *E* is the energy in eV, c the speed of light, s is the sensitivity in the diffraction mode, and *h* is the Planck constant in eV·s. Besides, the emission spectral curve can be deconvoluted into two Gaussians curves with center at ~1.35 and ~1.76 eV, with a full width at half-maximum (FWHM) of 0.28 and 0.24 eV, respectively. Moreover, a slight change in FWHM, and the value of peak energy was reported under an applied voltage. However, the exponential increment in emission intensity was also observed with the increment of bias voltage, as shown in Figure 13c. The phonon-assisted electroluminescence from SWNT and FLG was illustrated in Figure 13d. In addition, the phonons in the Γ-K direction with momentum ≤ 0.5|ΓK| is responsible for the momentum of electronic transition from M to K. Within this momentum range, it is the longitudinal optical (LO) branch, which is closest to Δ*E*/2 ≈ 0.2 eV, and hence most likely can provide the phonon involved in the emission process. In contrast, the TO branch with ~0.17 eV at a wave vector 0.5|ΓK| is slightly lower in energy [57]. The electron-phonon coupling (EPC) of those branches strongly depends on the position of the Fermi energy level E_F_ with respect to the charge neutrality point E_0_. At E_F_ = E_0_, the EPC for the LO and TO phonons is large only at the Γ and K points, respectively [164]. Lastly, all the electroluminescence emissions of graphene discussed in Section 4 are summarized in Table 3.

## 5. Conclusions and Outlook

Much interesting information has been put forward from recent progress in the field of graphene-based light emitters. Light emission has been reported from the different structures of graphene and related material, such as single/multi-layer graphene, reduced graphene oxide, and graphene-based heterostructures. Graphene light emitters may open the door to the development of mid-infrared to far-infrared light sources for gas sensing and infrared photodetection. The performance of light-emitting devices is highly influenced by different design parameters, such as ohmic contact, device, and material structure. The light emission radiations from graphene structures are explained in various theoretical aspects, including thermal emission, plasmons assisted emission, and electroluminescence, and have been extensively discussed. The wide range of emission spectrum has been demonstrated for visible to infrared wavelengths. Thermal emission from graphene follows a graybody/black body radiation curve, following Plank’s Law. Based on the review, none of the mechanisms can exactly explain the entire features of light emissions from graphene and related structures. 

Certain facts have also been extracted. Firstly, ionization/excitation is a conceivable mechanism to explain light emission from the graphene-based structure. The negative temperature coefficient is always accompanied by impact ionization, but the reported positive coefficient rules out the presence of impact ionization. Secondly, the EL emission peaks from graphene correspond to the local temperature (~4,500 K), if emission radiation is due to the Joule heating effect; this will nullify the light emission mechanism by thermal emission. In addition, in the electron tunneling effect, the charge carrier injection mechanism is responsible for the EL effect in the planner device, and light emissions are likely to occur at the drain terminal, which holds a wide bandgap. However, light emissions from the middle part of the graphene channel have also been addressed. The light emission from graphene supported on substrate localized at the center of the graphene channel and the position of the localized hot spot can be controlled by the source to drain or applied gate voltage. Some reports also addressed the fact that the emissions do not occur from the center of the graphene channel, and the V_G_ or V_DS_ cannot control the position of the light emission spot. Thus, the controlled position of the light emission point in the graphene channel is missing under applied voltage conditions; it excludes the presence of thermal emissions. 

Graphene has been the subject of intense research on optical and electronic applications. Therefore, numerous future challenges for the realization of graphene-based light emitters at a commercial level have been discussed. The maximum efficiency < 1% and short emission times from the suspended and substrate support graphene layer < 2 and 20 min, respectively, in vacuumed conditions are reported. However, in several other reports, the coated graphene layer with hexagonal boron nitride (h-BN) and aluminum trioxide (Al_2_O_3_) has shown an extended emission lifetime up to 1000 h. Further advancements in graphene-based light emitters can pave the way for highly efficient, flexible, transparent, compact, spectrally tunable light sources for future commercial applications. 

In order to improve the stability and performance of future graphene-based light emitters, the following aspects should be considered:

- The graphene-based active layer exhibits different optical, electrical, and chemical behavior. Oxidation of the graphene layer highly influences the efficiency, emission spectra, and lifetime of the graphene light-emitting device. The protective coating of the light-emitting graphene thin film possibly stops structural damage.
- By introducing an intersystem crossing path, singlet excitation could be achieved. The light source with an efficiency upper limit of up to 25% can be realized.
- Graphene-based doped material and nanocomposite material have not been investigated for light-emitting devices. As a possible solution, the doped graphene and graphene-based composite materials with numerous polymers could be used to tune the optical bandgap and surface plasmonic effect as well as thermal stability in graphene layer for optical emissions. Some critical issues in this emerging field of graphene-based light emitters, including short plasmons lifetime, instability of the induced bandgap, oxidation of the light active layer, low efficiency, and short lifetime, can also be overcome.
- Future demands for flexible optical and electronics require deformable and flexible light-emitting devices. Hence, future studies should be focused on the development of flexible graphene-based materials for optoelectronic devices.

## Figures and Tables

**Table 1:** Summary of the graphene-based thermal emitters.

Active Material	Deposition Method	Contact/Pattern Material	Patterning Method	Emission Mechanism	Substrate	Emission Spectrum	Authors and Reference
Graphene flakes	Mechanically exfoliation	Titanium/Palladium/Au lithographically	E-beam lithography and oxygen plasma etching	Joule heating effect	SiO_2_/Si	Infrared	Freitag et al. [45]
Graphene flakes	Mechanically exfoliation and CVD	Cr/Au electrodes Using microcontact transfer method for mechanically exfoliated graphene	E-beam lithography	Thermal radiation	Trench on SiO_2_/Si	Infrared to visible	Kim et al. [32]
Graphene	CVD Graphene	Cr/Au contact using thermal evaporation	E-beam lithography	Thermal emission	SiO_2_/Si	mid-infrared	Lawton et al. [46]
hBN/Graphene/hBN	Heterostructure (Van der Waals dry pickup method, using exfoliated Graphene flakes and hBN flakes)	Cr/Pd/Au, metal deposition	--	Thermal emission	SiO_2_/Si	Visible to near-infrared (400–1600 nm)	Kim et al. [96]
hBN encapsulated graphene layer	Mechanically exfoliation	Au contact	--	Thermal emission	SiO_2_/Si	mid-infra-red	Cheng et al. [104]
Graphene	Mechanically exfoliation and CVD) encapsulated with Al_2_O_3_	Ti/Pd	E-beam lithography	Thermal emission	SiO_2_/Si	Near-infrared	Miyoshi et al. [62]

**Table 2:** The summary of plasmons-assisted emissions from graphene.

Active Material	Deposition Method	Contact/Pattern Material	Patterning Method	Emission Mechanism	Substrate	Emission Spectrum	Reference
Graphene sheet	Theoretical model			Electron-plasmons interaction	Dielectric	Soft and hard X-ray radiation	Liang et al. [116]
Graphene	Theoretical model			Quantum Čerenkov effect	-	terahertz to near-infrared	Ido Kaminer et al. [55]
Graphene	Mechanical exfoliation	Au physical vapor deposition	Wire-based shadow masking	Quantum Čerenkov effect	SiO2/Si	visible and near-infrared	A. Beltaos el al. [76]

**Table 3:** The summary of electroluminescence (EL) emissions.

Active Material	Deposition Method	Contact Pattern Material	Patterning Method	Emission Mechanism	Substrate	Emission Spectrum	Authors and Reference
Graphene	Mechanical Exfoliation	STM Analysis, using Au Tips		Hot EL	Glass Coated with ITO	~550 To 900 nm	Beam et al. [58].
Semi Reduced Graphene Oxide	Drop Casting	Light Scribe		Poole Frankel’s Emission	PET	~450 To 750 nm	Wang et al. [63]
GNRs	Synthesized On Au Surface	STM Analysis, Using Au Tips		EL	Au (111)	Narrow Band, Red Light Emission	Chong et al. [140]
FLG, SWNT, and MWNT	Dielectrophoretic Deposition	Pd, Au/Cr	E Beam Lithography	Phonon Assisted EL	Si/SiO_2_	Visible Spectrum 1.4 And 1.8 eV Peaks	Essig et al. [57]
